# Adjustment and Characterization of an Original Model of Chronic Ischemic Heart Failure in Pig

**DOI:** 10.4061/2010/542451

**Published:** 2010-09-07

**Authors:** Laurent Barandon, Joachim Calderon, Patricia Réant, Dominique Caillaud, Stéphane Lafitte, Xavier Roques, Thierry Couffinhal, Pierre Dos Santos

**Affiliations:** ^1^Inserm U 828, Avenue du Haut-Lévêque, 33600 Pessac, France; ^2^Department of Cardiac Surgery and Anesthesiology, Haut-Lévêque Hospital, 33600 Pessac, France; ^3^University of Medicine, Bordeaux 2, rue Léo Saignat, 33000 Bordeaux, France; ^4^Technological Platform for Biomedical Innovation, avenue du Haut-Lévêque, 33600 Pessac, France; ^5^Laboratory of Echocardiography, Haut-Lévêque Hospital, 33600 Pessac, France

## Abstract

We present and characterize an original experimental model to create a chronic ischemic heart failure in pig. Two ameroid constrictors were placed around the LAD and the circumflex artery. Two months after surgery, pigs presented a poor LV function associated with a severe mitral valve insufficiency. Echocardiography analysis showed substantial anomalies in radial and circumferential deformations, both on the anterior and lateral surface of the heart. These anomalies in function were coupled with anomalies of perfusion observed in echocardiography after injection of contrast medium. No demonstration of myocardial infarction was observed with histological analysis. Our findings suggest that we were able to create and to stabilize a chronic ischemic heart failure model in the pig. This model represents a useful tool for the development of new medical or surgical treatment in this field.

## 1. Introduction

Ischemic cardiomyopathy plays a predominant part in the etiology of patients with heart failure [1]. Despite the progress in various types of treatment, despite the improvement in many percutaneous revascularization techniques [2], and despite the progress in coronary artery bypass surgery [3], the cardiac surgeon is presented with many patients with chronic ischemia who cannot benefit from revascularization techniques. Patients with heart failure require frequent hospitalisation with a substantial rate of mortality and represent a high cost to society [4, 5]. Cardiac surgeons take an ever greater leading role in the management of these patients with the aim of trying to reduce the morbidity and mortality rates. The development of experimental models close to the clinical reality of a patient with heart failure is a major approach to a better understanding of the pathophysiological mechanisms set in motion. This will take part in improving essential knowledge with regard to heart failure, to imagine new therapeutic concepts, whether medical or surgical. In the literature, there are numerous models of heart failure, whether the result of genetic modification [6], of a permanent ligature of the coronary arteries with, or without, reperfusion (models of infarction and not of ischemia) [7–9], of electrical stimulation at a rapid rate [10], of modifications of load [11], or of toxic origin [12, 13]. On the other hand, some models have been developed to induce myocardial ischemia without heart failure [14]. These various models have enabled us to improve our knowledge, but they do not go far enough in meeting the clinical reality of a patient with heart failure and ischemia. The aim of our study was to develop a model for chronic ischemic heart failure which was stable and reproducible. To be able to represent an interface and a tool useable by all the protagonists gravitating around this disease and to position it in the preclinical period, the model was set up in pigs. We present the development of a model for chronic heart failure by double constriction of the coronary artery (circumflex branch of left coronary artery and anterior descending branch) responsible for progressive stenoses.

## 2. Material and Methods

### 2.1. Animals

Male pigs, 2 months aged weighing 15 to 20 kg were used for this procedure. These animals came from an animal supply facility where they are housed pre- and postoperatively and where the rules of good practice with regard to the management of animals for research are met. This study was conducted in accordance with both institutional guidelines and those in force in the European community for experimental animal use (L358–86/609/EEC).

### 2.2. Design of Study

**Figure 1:**
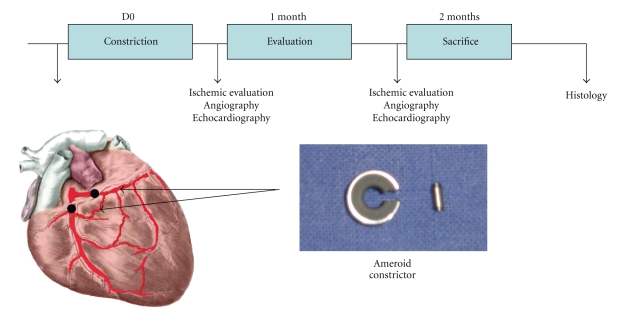
Study design.

Details of the design are given in Figure 1. Briefly, a first echocardiography was performed just before the surgery (baseline). The follow-up was targeted at one and two months after the procedure. Parameters as well as Left Ventricle (LV) function, LV geometry, wall motion score, thickening, circumferential and longitudinal strain, and analysis of the myocardial perfusion were stored and compared.

### 2.3. Preparation and Anaesthesia

The animals were pre-medicated with an intramuscular injection of Calmivet (Acepromazine, 0.5 mL). They were then transferred to the operating room. A catheter was placed in the vein at the level of the left ear (cathlon 22 gauge) which enabled induction of anaesthesia, combining Imalgène (30 mg/kg-ketamine) and Rompun (1 mL/kg-xylazine). The pig was then intubated with a no. 7 reinforced tube using a large right blade laryngoscope. Ventilatory parameters were as follows: frequency 15–20/min, Volume 8–10 mL/kg. The pig was placed in the dorsal recumbent position. Homeostasis was maintained using a heated operating table, controlled by a temperature probe.

### 2.4. Heart Failure Model: Double Constriction

**Figure 2:**
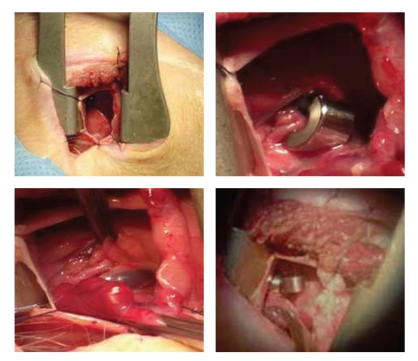
After incision in the thorax in the 4-5th intercostal space, the pericardium is opened. The left atrium is reclined, the atrioventricular groove dissected, and the circumflex branch of left coronary artery is exposed. The constrictor is placed around it. A second constrictor is then placed around the left anterior descending artery.

An incision was made into the thorax at the level of the left fifth intercostal space. The pericardium was opened radially on contact with the left atrium. This was then reclined, the dissection continuing towards the dihedral angle between the atrium and the pulmonary artery. The distal part of the left trunk then appeared as well as the division between the circumflex coronary artery and anterior descending branch. The proximal circumflex coronary artery was then exposed and the first ameroid constrictor placed around it (3.5 mm, Research Instruments, Lebanon, Oregon) just above the first lateral branch (Figure 2). The descending branch was exposed, then a second constrictor of the same type was placed just below the first septal branch (Figure 2). After closing up the pericardium, the ribs were closed surgically. A pleural exsufflation and a positive expiratory pressure enabled air to be emptied and to remove atelectasis in the left lung. The muscle wall was closed after injection of a local anaesthetic, such as Naropeine. An intravenous infusion of an analgesic (100 mg of ketoprofen) was administered as well as an injection of aspirin (200 mg) to activate antiplatelet treatment. This treatment was continued *per os* for 10 days in association with Cordarone (200 mg/day).

### 2.5. Evaluation by Echocardiography

A Vivid 7 (GE Medical Systems, Horten, Norway) was used to acquire echocardiographic data with a 4-MHz transducer. All the evaluations were performed at rest. 

To determine the percentage of thickening, the method was performed using the following formula TeleDiastolic Diameter—TeleSystolic Diameter/TeleDiastolic Diameter. Results were expressed in percentage. 

To study Wall motion score, the LV was divided into 12 segments assigned to coronary artery supply. Image analysis was performed by an experienced echocardiographer unaware of swine disease, who analyzed stored 2D cine loops in random order. Quad-screen display was used to view the 2D parasternal long-axis and short-axis views and apical 4-chamber, 2-chamber, and long-axis views at baseline. Wall motion score was assessed using a side-by-side visual assessment. The development of new or worsening resting LV wall motion abnormalities in >1 contiguous segment of the same vascular territory was considered a sign of inducible ischemia. Segmental wall motion was scored as follows: 1 = normal or hyperkinetic, 2 = hypokinetic, 3 = akinetic, and 4 = dyskinetic. 

For myocardial function, a conventional echocardiogram of the ejection fraction was carried out. Briefly, the Simpson's formula was used (TeleDiastolic Volume—TeleSystolic Volume/TeleDiastolic Volume) and results were expressed in percentage.

To study myocardial perfusion, we used contrast echocardiography **at** **rest** with calculation of perfusion flow-rate in real-time mode together with a flash sequence. A microbubble solution of the type Sonovue* (BRACCO) was given by central intravenous infusion to show the level of perfusion at the level of the myocardial wall [15]. Myocardial signal intensity (SI) was measured on end-systolic images at each pulsing interval. Three transmural myocardial regions of interest that encompassed the anterolateral, the septal, and the inferior wall were selected. The SI was determined in these regions of interest and averaged from 5 images for each incremental delay. SI at every frame was fitted to an exponential function, *y* = *A*(1 − *e* ^−*bt*^), where *y* is SI at any given time, *A* is the SI plateau that reflects microvascular cross-sectional area or myocardial blood volume, and *b* is the slope of the refilling curve that reflects myocardial microbubble velocity. The product of *A* and *b* (*A*∗*b*) reflects the myocardial blood flow.

Tools for quantifying 2-dimensional strain (speckle tracking) enabled us, through a recording of conventional black and white images in the various zones, to obtain the parameters of myocardial deformation. These deformations depending on the orientation of acquisitions were expressed in the form of circumferential, longitudinal, and radial components closest to the orientation of the myocardial fibres. These components were exploited at rest as described in [16]. The deformation data were obtained by automatic measurement of the distance between 2 pixels of an LV segment during the cardiac cycle and are independent of angle. The data were stored and transferred to a computer for postprocessing analyses. The recordings were analyzed with Echopac software (GE Medical Systems).

### 2.6. Evaluation by Coronary Angiography

 Coronary angiography was used to monitor the progressive evolution of stenosis of the coronary arteries, to assess the intensity of flow through the stenoses, and to check collaterality from the right coronary artery. It was carried out through the femoral artery using a 4 Fr. catheter.

### 2.7. Histological Analysis

After sacrifice, various fragments from the anterior, lateral ischemic zones, as well as the healthy zones were harvested, then fixed in methanol as well as cryopreserved. These various fragments were stained with Masson's trichrome stain. Analysis of inflammatory reaction was carried out by labelling with myeloperoxydase, with CD 45, and with CD 3. Capillary density was performed by immunostaining of endothelial cells with CD 31 [17].

### 2.8. Statistical Analysis

The results are expressed in mean ± standard deviation. All the analyses were carried out using the relevant software (Statview 5–1). Comparisons of the continuous variables between 2 groups were carried out using ANOVA. If a statistical difference was found, a *t*-test was carried out. A value of *P* < .05 was considered to be significant.

## 3. Results

### 3.1. Mortality

Twenty-seven pigs were operated for this study. Two died during the procedure for implanting the constrictors (1 from a tear in the left atrium, 1 from ventricular fibrillation). Ten died during the follow-up period. Six died (25%) from congestive heart failure and 4 died from cardiac rhythm disorder (probably VT, 16%) caused suddenly. We were able to assess 15 animals for the present study. Total mortality rate was 40%. In a preliminary study and in the absence of programmed sacrifice (only with the aim of studying survival in the longer term), all the animals died at three months.

### 3.2. Assessment of Stenoses

The degree of stenosis was assessed by immediate postoperative coronary angiography, and at 1 and 2 months. No stenosis was seen in the immediate postoperative examination. At 1 month, the stenoses observed both on the circumflex and on the anterior descending branch were approximately 90%. The flow was scored TIMI 2, except for 1 case where the circumflex was thrombosed. Similar results were observed at 2 months with no worsening of the already existing stenoses. In contrast, in our hand, we were never able to demonstrate any collateralization on the follow-up angiographies with a specific analysis from the right coronary artery.

### 3.3. Assessment of Cardiac Function and Myocardial Ischemia

**Figure 3:**
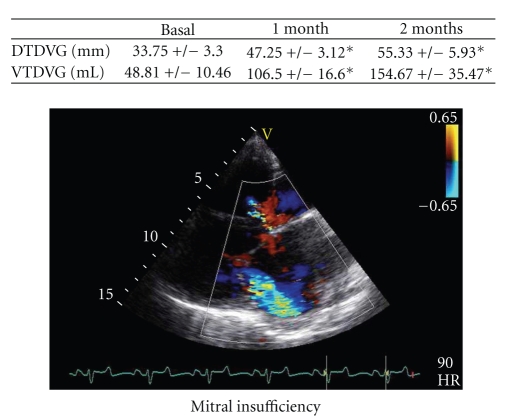
One to 2 months after constriction, the left ventricle dilates and a significant mitral insufficiency appears.

**Figure 4:**
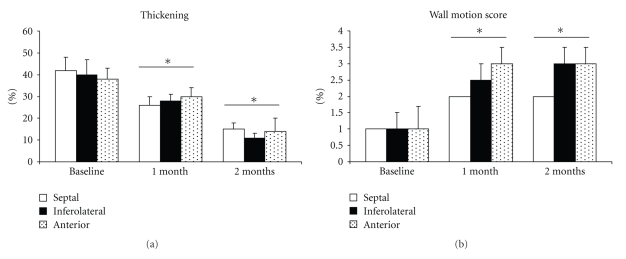
Over time, a substantial decrease in the thickening and the mean wall motion score of ischemic myocardial walls can be observed; *P < .01 compared to basal time.

**Figure 5:**
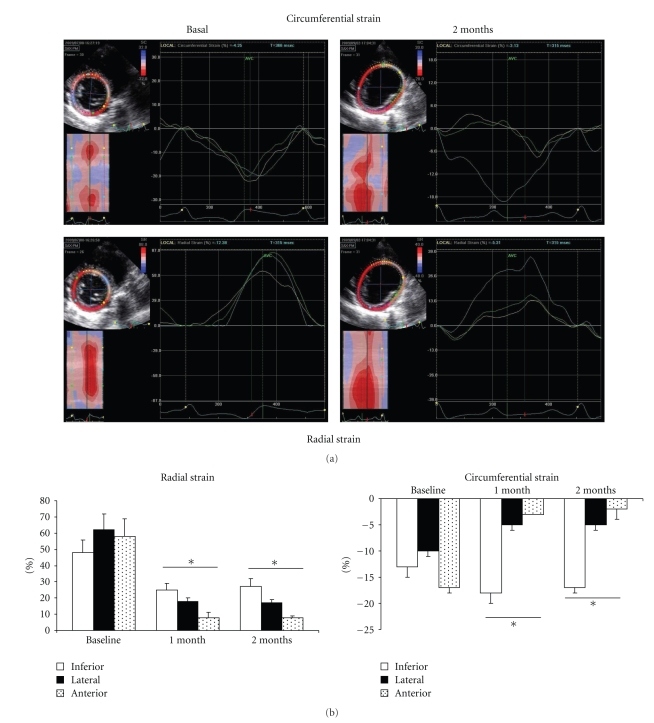
(a) Echocardiographic visualisation of the development of radial and circumferential deformation in healthy and ischemic zones. Quantification of peaks in circumferential and radial strain in ischemic and control areas showed substantial anomalies in radial and circumferential deformations alike, both on the anterior and lateral surface of the heart, with compensation marks on the inferior healthy surface (*P < .01 compared to basal time). (b) Graphic representations of anomalies in radial and circumferential deformation in walls of myocardium.

**Figure 6:**
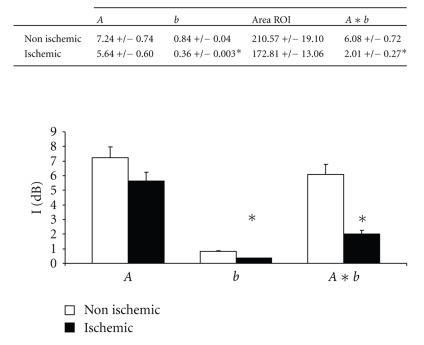
Echocardiographic evidence of defects in perfusion after injection of contrast medium. As shown, myocardial blood flow was significantly decrease in the anterior and lateral areas (*P  < .05) compared to the healthy zone (inferior wall of the left ventricle).

At 1 month, with identical results at 2 months, there was significant alteration in left ventricular function of around 30% to 35%. As shown in Table 1, this dysfunction was accompanied by important modifications of the LV geometry and dilation (as well as in LVTDV, LVTDD, E/Ea, Left Atrial size, wall thickness; Table 1) and by significant mitral insufficiency (grade III/IV) where the mechanism was mixed, because of restriction of movement of the posterior mitral valve and by annular dilation (Figure 3, *P <.05). A significant decrease in percentage of thickening (Figure 4, 38 ± 5% at basal time to 30 ± 4% at 1 month and to 14 ± 6% at 2 months after constriction in the anterior zone, *P < .01) as well as substantial alteration of the mean wall motion score was found (Figure 4, 1 ± 0.7 at basal time to 3 ± 0.5 at 1 or 2 months after constriction in anterior area with similar results in inferolateral zone, *P < .01 compared to basal time). The circumferential and radial strain were obtained in the parasternal short-axis view. Apical views were used to measure longitudinal strain as well as in the anterior, lateral, and inferior wall. Echocardiographic analysis consisted of measurement of the peak of strain in the end-systolic phase within the ischemic and the healthy zone (Figure 5(a)). Quantification of peaks (white arrows) in circumferential strain in end systole in ischemic areas (yellow curve) and control areas (blue curve) showed substantial anomalies in radial (e.g., 58 ± 11% at basal time compared to 8 ± 1% at 2 months in the anterior area, *P < .01) and circumferential deformations (e.g., −17 ± 1% at basal time compared to −2 ± 2% at 2 months in the anterior area, *P < .01) alike, both on the anterior and lateral surface of the heart, with compensation marks on the inferior (or healthy) surface (e.g., in circumferential strain −13 ± 2% at basal time compared to −18 ± 2% at 2 months in the anterior area, *P < .01, Figures 5(a) and 5(b), *P < .01). These anomalies in function were coupled with anomalies of perfusion observed in echocardiography after injection of contrast medium. As shown in Figure 6, there were perfusion defects in the anterior and lateral areas (*P <.05) compared to the healthy zone (inferior wall of the left ventricle) corresponding in a significant decrease in myocardial blood flow.

### 3.4. Histological Assessment of the Ischemic Heart

**Figure 7:**
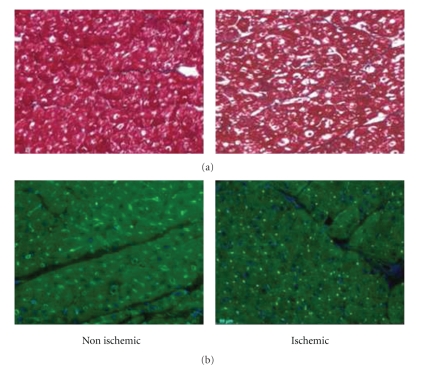
(a) Staining with Masson's trichrome stain comparing a healthy zone (inferior wall of the left ventricle) with an ischemic zone (anterior wall with similar results for lateral wall). No necrosis was found demonstrating the absence of myocardial infarction. (b) Immunostaining with fluorescence of lectin found significant reduction in capillary density in anterior compared to the inferior wall.

After sacrifice, the hearts were collected for histological analysis. Staining with Masson's trichrome stain enabled elimination of the presence of infarction (Figure 7(a)). Immunostaining of vessels (anti-CD 31, lectin) found a significant reduction in capillary density at the level of the anterior and lateral surfaces compared with the healthy zone (Figure 7(b)) of approximately 30%. Immunostaining for inflammatory cells in the ischemic zones found a moderate inflammatory response with few CD 45 positive cells in the anterior and inferior wall. 

## 4. Discussion

Ischemic heart failure is a major cause of morbidity and mortality in developed countries. Much progress has been made, both in understanding the pathophysiological processes involved and in the medical and surgical management of these patients. However, many problems remain and require the development of new concepts to improve the prognosis for our patients. In this sense, cardiac surgeons, in association with all the actors in health and research who are involved in heart failure, play an essential role in trying to manage this disease better. Developing experimental models for ischemic heart failure is an important part in the progression of research in this field. Our object was to develop and define a preclinical model for ischemic heart failure in a large animal. Our choice focused on the pig because of the possible extensions for surgical treatment which could be developed. In the literature, there is no model, to date, for heart failure secondary to chronic ischemia. Many models have been developed with the aim of making a ventricle deficient but they do not pertain to a process of chronic ischemia. Whether they are models for infarction by ligature, by embolization, by endocoronary balloon, by injection with toxins, or by rapid rhythm electrical stimulization, permanent myocardial ischemia cannot be superimposed on any of them [18]. In our unit, we developed a model for chronic myocardial ischemia by constriction (ameroid constrictor) of the circumflex branch of left coronary artery [19]. This model, already used by many research centres, offers the possibility of creating ischemia in the inferior-lateral wall of the left ventricle. Although interesting for many fields, such as cell therapy, for example, [20–22], it does not provide left ventricle dysfunction and cannot therefore be used in the field of heart failure. We hypothesized that creating severe ischemia both in the anterior and in the inferior-lateral region might be a good strategy for developing heart failure. We developed and defined the model for chronic ischemic heart failure by double constriction. This model is viable and reproducible despite significant mortality. Chronic constriction has the advantage of building up progressively with the growth of the animal. Histological analysis enabled us to avoid any underlying necrosis thus eliminating dysfunction due to infarction. As evidence, the myocardium is viable and the moderate inflammatory response observed in the area at risk probably help for angiogenesis. The tools required for assessing our model were based firstly on coronary angiography, which showed us anatomically the progression of the stenoses. Interestingly, we were never able to demonstrate any collaterality from the right coronary artery. In this way, we could not support any compensation from the right coronary artery in regional or global function. The second tool for assessment was based on echocardiography, at rest by analysis of perfusion and deformation. We demonstrated that the myocardium presented a substantial alteration of perfusion but this remains persistent. These defects in perfusion were accompanied by morphometric modifications in the left ventricle with dilation and without the appearance of myocardial infarction. There were anomalies in the deformation of the ventricular walls in the 3-dimensional areas in the ischemic zones. All these elements are responsible for severe left ventricular dysfunction. This experimental study was also designed to find out if assessment of 2-dimensional strain could detect contraction abnormalities induced by ischemia in a severe model of heart failure. Our results suggested that 2-dimensional strain was reliable for detection of myocardial contraction abnormalities under ischemic conditions at rest at an earlier stage.

However, the study presented some several limitations. First at all, we observed an important rate of mortality during the follow-up. This mortality was probably dependant on the model but this high incidence of sudden death or end stage heart failure represented a severe limitation to improve our understanding of pathophysiology. Second, we have not performed hemodynamic study and we could not evaluate LVEDP which would be an important target in this model. The LV filling pressures were only analyzed echographycally with the ratio E/Ea. Finally, all the measurements were performed at rest without dobutamine infusion. Histological analysis had never demonstrated necrosis and myocardial infarction in the area at risk traducing the anatomical integrity of the myocardial wall (viable myocardium). If 2-dimensional strain analysis has clearly demonstrated its role in the evaluation of our model at rest, our results could be strengthened at stress.

We have therefore created a stable model for ischemic heart failure and developed tools to define it. This model could be used to assess new medications, new procedures for echocardiographic analysis, to implement new procedures for biventricular stimulation, or more specifically for surgeons as a basis for new techniques for surgical management of ischemic heart failure.

## Conflict of Interests

All the authors do not have a direct financial relation with the commercial identities mentioned in the present paper that might lead to a conflict of interests.

## Figures and Tables

**Table 1:** Morphometric and functional analysis at basal time and during the follow-up. LVTDD: Left Ventricle TeleDiastolic Dimension. LVTDV: Left Ventricle TeleDiastolic Volume, dIVSW: diastolic InterVentricular Septal Wall, dPLVW: diastolic Posterior Left Ventricle Wall, E/Ea: Peak mitral inflow velocity—E- and Early diastolic—Ea-peak myocardial velocities with Tissue Doppler Imaging, LAV: Left Atrial Volume, WMS ant: Anterior Wall Motion Score, WMS inf: Inferior Wall Motion Score, and WMS Sept: Septal Wall Motion Score. Data are in mean ± SD. *P < .05, **P < .01 compared to basal time.

	Basal Time	1 month	2 months
LVTDD (mm)	33.75 ± 3.3	47.25 ± 3.12*	55.33 ± 5.93**
LVTDV (mL)	48.81 ± 10.46	106.5 ± 16.6*	154.67 ± 35.47**
dIVSW (mm)	7.73 ± 0.24	7.81 ± 1.2	8.67 ± 0.24*
dPLVW (mm)	6.09 ± 0.21	7.38 ± 0.9*	7.53 ± 0.47*
E/Ea	4 ± 0.3	7 ± 0.32*	9 ± 0.56*
LAV (cm^2^)	7.5 ± 2.06	12.30 ± 2.48*	16.84 ± 3.5**
WMS ant	1 ± 0.7	3 ± 0.5*	3 ± 0.5*
WMS inf	1 ± 0.4	2.5 ± 0.6*	3 ± 0.5*
WMS Sept	1	2	2

## References

[1] Hunt SA, Abraham WT, Chin MH (2009). 2009 focused update incorporated into the ACC/AHA 2005 guidelines for the diagnosis and management of heart failure in adults: a report of the Aamerican College of Cardiology Foundation/American Heart Association Task Force on practice guidelines: developed in collaboration with the International Society for Heart and Lung Transplantation. *Circulation*.

[2] Friedrich GJ, Bonatti J (2007). Hybrid coronary artery revascularization—review and update 2007. *Heart Surgery Forum*.

[3] Eagle KA, Guyton RA, Davidoff R (2004). ACC/AHA 2004 guideline update for coronary artery bypass graft surgery: summary article. A report of the American College of Cardiology/American Heart Association Task Force on Practice Guidelines (Committee to Update the 1999 Guidelines for Coronary Artery Bypass Graft Surgery). *Circulation*.

[4] de Lissovoy G, Fraeman K, Salon J, Chay Woodward T, Sterz R (2008). The costs of treating acute heart failure: an economic analysis of the SURVIVE trial. *Journal of Medical Economics*.

[5] de Lissovoy G, Fraeman K, Teerlink JR (2009). Hospital costs for treatment of acute heart failure: economic analysis of the REVIVE II study. *European Journal of Health Economics*.

[6] Yamada S, Nelson TJ, Crespo-Diaz RJ (2008). Embryonic stem cell therapy of heart failure in genetic cardiomyopathy. *Stem Cells*.

[7] Bjerre M, Jensen H, Andersen JD (2008). Chronic ischemic mitral regurgitation induced in pigs by catheter-based coronary artery occlusion. *The Journal of Heart Valve Disease*.

[8] Krombach GA, Kinzel S, Mahnken AH, Günther RW, Buecker A (2005). Minimally invasive close-chest method for creating reperfused or occlusive myocardial infarction in swine. *Investigative Radiology*.

[9] Schmitto JD, Coskun KO, Coskun ST (2009). Hemodynamic changes in a model of chronic heart failure induced by multiple sequential coronary microembolization in sheep. *Artificial Organs*.

[10] Zhou S-X, Lei J, Fang C, Zhang Y-L, Wang J-F (2009). Ventricular electrophysiology in congestive heart failure and its correlation with heart rate variability and baroreflex sensitivity: a canine model study. *Europace*.

[11] Donal E, Bergerot C, Thibault H (2009). Influence of afterload on left ventricular radial and longitudinal systolic functions: a two-dimensional strain imaging study. *European Journal of Echocardiography*.

[12] Goetzenich A, Hatam N, Zernecke A (2009). Alteration of matrix metalloproteinases in selective left ventricular adriamycin-induced cardiomyopathy in the pig. *Journal of Heart and Lung Transplantation*.

[13] Borenstein N, Bruneval P, Behr L (2006). An ovine model of chronic heart failure: echocardiographic and tissue Doppler imaging characterization. *Journal of Cardiac Surgery*.

[14] Fallavollita JA, Perry BJ, Canty JM (1997). 18F-2-deoxyglucose deposition and regional flow in pigs with chronically dysfunctional myocardium: evidence for transmural variations in chronic hibernating myocardium. *Circulation*.

[15] Reant P, Labrousse L, Lafitte S (2008). Experimental validation of circumferential, longitudinal, and radial 2-dimensional strain during dobutamine stress echocardiography in ischemic conditions. *Journal of the American College of Cardiology*.

[16] Lafitte ST, Higashiyama A, Masugata H (2002). Contrast echocardiography can assess risk area and infarct size during coronary occlusion and reperfusion: experimental validation. *Journal of the American College of Cardiology*.

[17] Barandon L, Couffinhal T, Ezan J (2003). Reduction of infarct size and prevention of cardiac rupture in transgenic mice overexpressing FrzA. *Circulation*.

[18] Geens JH, Trenson S, Rega FR, Verbeken EK, Meyns BP (2009). Ovine models for chronic heart failure. *International Journal of Artificial Organs*.

[19] Radke PW, Heinl-Green A, Frass OM (2006). Evaluation of the porcine ameroid constrictor model of myocardial ischemia for therapeutic angiogenesis studies. *Endothelium*.

[20] Quevedo HC, Hatzistergos KE, Oskouei BN (2009). Allogeneic mesenchymal stem cells restore cardiac function in chronic ischemic cardiomyopathy via trilineage differentiating capacity. *Proceedings of the National Academy of Sciences of the United States of America*.

[21] Schneider C, Krause K, Jaquet K (2008). Intramyocardial transplantation of bone marrow-derived stem cells: ultrasonic strain rate imaging in a model of hibernating myocardium. *Journal of Cardiac Failure*.

[22] Schuleri KH, Feigenbaum GS, Centola M (2009). Autologous mesenchymal stem cells produce reverse remodelling in chronic ischaemic cardiomyopathy. *European Heart Journal*.

